# Entirely Off-Grid and Solar-Powered DNA Sequencing of Microbial Communities during an Ice Cap Traverse Expedition

**DOI:** 10.3390/genes10110902

**Published:** 2019-11-07

**Authors:** Glen-Oliver. F. Gowers, Oliver Vince, John-Henry Charles, Ingeborg Klarenberg, Tom Ellis, Arwyn Edwards

**Affiliations:** 1 Vatnajökull Expedition Team, UK; oliver.vince94@gmail.com (O.V.); johnhenry.charles@gmail.com (J.-H.C.); 2 Imperial College Centre for Synthetic Biology (IC-CSynB), Imperial College London, London SW7 2AZ, UK; t.ellis@imperial.ac.uk; 3 Department of Bioengineering, Imperial College London, London SW7 2AZ, UK; 4 Institute of Biomedical Engineering, University of Oxford, Oxford OX3 7DQ, UK; 5 Faculty of Natural Resource Science, University of Akureyri, 600 Akureyri, Iceland; ingeborg@unak.is; 6 Faculty of Life and Environmental Sciences, University of Iceland, 101 Reykjavík, Iceland; 7 Institute of Biological, Environmental & Rural Sciences (IBERS), Aberystwyth University, Aberystwyth SY23 3DD, UK; aye@aber.ac.uk; 8 Interdisciplinary Centre for Environmental Microbiology, Aberystwyth University, Aberystwyth SY23 3DD, UK

**Keywords:** metagenomics, nanopore, polar, expedition, microbial sequencing

## Abstract

Microbial communities in remote locations remain under-studied. This is particularly true on glaciers and icecaps, which cover approximately 11% of the Earth’s surface. The principal reason for this is the inaccessibility of most of these areas due to their extreme isolation and challenging environmental conditions. While remote research stations have significantly lowered the barrier to studying the microbial communities on icecaps, their use has led to a bias for data collection in the near vicinity of these institutions. Here, miniaturisation of a DNA sequencing lab suitable for off-grid metagenomic studies is demonstrated. Using human power alone, this lab was transported across Europe’s largest ice cap (Vatnajökull, Iceland) by ski and sledge. After 11 days of unsupported polar-style travel, a metagenomic study of a geothermal hot spring gorge was conducted on the remote northern edge of the ice cap. This tent-based metagenomic study resulted in over 24 h of Nanopore sequencing, powered by solar power alone. This study demonstrates the ability to conduct DNA sequencing in remote locations, far from civilised resources (mechanised transport, external power supply, internet connection, etc.), whilst greatly reducing the time from sample collection to data acquisition.

## 1. Introduction

As the Earth’s climate continues to warm, the role that microbial ecosystems play in anthropogenic climate change is becoming increasingly apparent. As these microbial communities affect the climate, they, too, will be affected [1] and may further amplify climate change [2]. It is crucial that our understanding of this interaction improves to inform our collective knowledge of how climate is changing [1]. Our understanding of these microbial communities naturally correlates well with the ease of access for sample collection [3,4]. In particular, polar environments suffer from logistically challenging sample collection and shipping. Hence, polar environments remain poorly understood at this microbial level despite the polar cryosphere representing approximately 14% of the Earth’s surface [5]. Traditionally, samples must be collected on site using sterile equipment and transported back to a laboratory under appropriate conditions, such as under dry ice. Logistics are further hampered by clearing customs and other delays in transit. This often results in a significant length of time from sample collection to sample analysis. During this time, the composition of the microbial community can change or degrade. 

One solution to these issues is to bring the laboratory closer to the sample collection site. This has been achieved by building remote institutions and research stations, for example networks of Arctic stations (https://eu-interact.org/) and the many research stations on the Antarctic continent [6]. Whilst this minimises the time and logistic requirements of sample analysis, this benefit is greatest for study sites in proximity to field stations [7]. For example, a recent meta-analysis showed that 31% of Arctic ecology citations are derived from work within a 50 km radius of just two field stations [7].

Conventional characterisation of microbial communities relies on DNA sequencing, since most microbes remain uncultured [8]. Both high throughput metagenomic sequencing and amplicon sequencing are typically applied with the intention of revealing the proportions of microbial species present in a particular sample [5,9,10]. Over the last decade, short read sequencing has been used predominantly. This requires a full genomics laboratory to house large, bulky equipment. In recent years, long read sequencing has seen increased use. Oxford Nanopore sequencing can produce significant amounts of read data (Mbp—Gbp) from a small device capable of being run directly from a laptop [11]. Previous studies have capitalised on the small-form factor of this device to conduct sequencing efforts at the site of sample collection. For example, portable long-read sequencing was used to sequence plant virus genomes in real time to inform crop management in sub-Saharan Africa [12]. It has also been proven to provide early detection and epidemiologic surveillance of Ebola and Zika virus’ during epidemic outbreaks [13,14]. The clear advantage of this technology is that the device can be run in low-resource environments. This is largely because sample preparation requires a minimal molecular biology set up and the sequencing run itself requires only a laptop, a Nanopore MinION device, and a Nanopore flowcell. This device has also been taken underground to demonstrate the ability to sequence at inhospitable sub-surface levels [15]. It is a clear progression to then apply these minimal-infrastructure techniques to polar environments. Temperature regulation is a key challenge in these environments as nanopore flowcells are extremely sensitive to freezing. Teams have previously conducted DNA sequencing in polar environments using Oxford Nanopore devices. Johnson et al. conducted on-site calibrations in the Antarctic Dry Valleys and further sequencing from samples at a research station [16]. Edwards et al. similarly performed sequencing at an Arctic research station following sample collection nearby [10]. Edwards et al. further successfully achieved in-field sample-to-data collection at a field camp in Greenland (67° N) using a combination of a generator and battery power that was flown in [10]. 

Thus far, the ability to sequence DNA off-grid has been limited by access to vehicles and generators. This study aimed to minimise the infrastructure required for off-grid DNA sequencing by utilising solar power alone for all DNA extraction and sequencing in a polar environment. Due to both long days in the summer and reflective ice and snow, solar energy is in no short supply in polar environments, and therefore provides the perfect compromise between portability and renewable power availability. This demonstrates that a sequencing effort could be successfully completed midway through an ongoing, unsupported expedition alongside the other physical and psychological challenges associated with survival in such a challenging environment. Data collection was conducted in a tent after the expedition team had conducted a ski traverse of Europe’s largest ice cap (100 km) over 11 days and used solar power alone. This study occurred at least 60 km from the nearest civilisation and at least 135 km from the nearest research facility. Crucially, everything required to conduct this metagenomic study was transported in a sledge along with all equipment required for long-term polar survival. 

## 2. Methods

### 2.1. Hardware Logistics

The entire laboratory setup was contained within two 9 L boxes in addition to a laptop (in waterproof/shockproof case) and 90 W solar panel (Table 1). This was packed into the back of a 50 kg rated polar sledge (Aguille Alpine Trail Pulk, Cumbria, UK) and occupied ~30% of the sledge volume.

**Figure 1:**
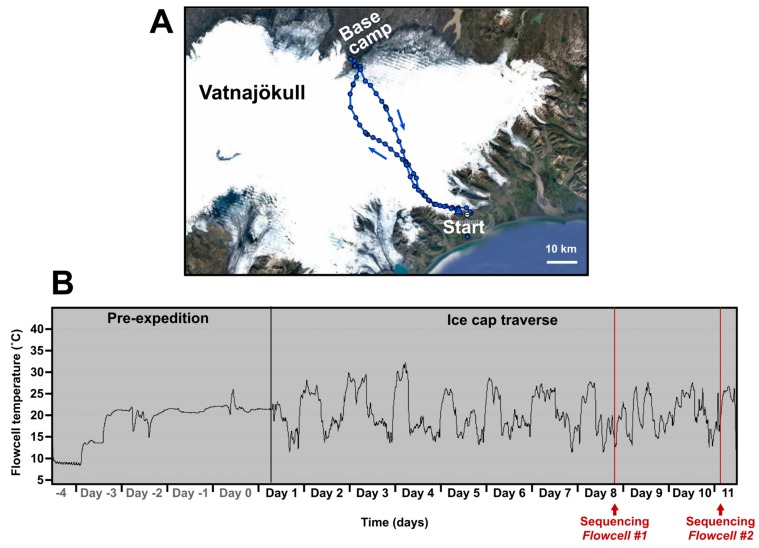
Flowcells were maintained above freezing using body heat for the outbound eleven ski days. (**A**) Pulks (including the DNA sequencing equipment) were pulled by ski for seven days on the outbound journey to the “Base camp” on the northern edge of the ice cap. The return journey was reduced to four days due to more favourable ski and weather conditions. (**B**) Temperature log of the flowcells from leaving the UK to the start of the second sequencing run. During Day −4 the flowcells were kept in a polystyrene box with cool packs equilibrated to 4 °C and put in the hold during the flight. Days −3 to 0 were spent travelling to and staying at a hostel where flowcells were kept indoors at room temperature. Days 1 to 7 were spent traversing the icecap. Spikes in temperature correspond to night time where flowcells were kept inside the sleeping bag. Days 8 to 11 were spent at “Base camp” on the northern edge of the ice cap. Sequencing runs #1 and #2 (red lines) occurred at the base camp on Day 8 and Day 11.

During the flight to Iceland, kit was stored in three different conditions: [cargo] Hold (ambient), Hold (polystyrene box with 4 °C ice packs), and hand-luggage (Table S1). During the ice cap traverse (route shown in Figure 1A) three temperature zones were also established, kept on person (“Chest—warm”), taken into the tent each night (“Tent—box”), and left outside in the pulk (“Pulk—box”) (Table S1). The temperature environment of “Chest—warm”, importantly, contained the nanopore flowcells that are destroyed if allowed to freeze. To control the temperature of the flowcells they were kept in the sleeping bag at night inside a Peli™ Case 1015 (Peli Products Ltd, Glossop, UK) and during the day items were kept inside a generic airtight plastic food container with foam padding for thermal insulation. This case was kept in a travel bag with a strap around the carrier’s neck. Temperature of the flowcells was constantly recorded (“Hold (polystyrene box)” during flight and “Chest-warm” during ski traverse) using a battery powered automatic temperature logger. This method maintained a temperature between ~+9 °C and ~+30 °C despite ambient outdoor temperatures reaching −17 °C (Figure 1B). Overheating during exercise on warmer days became a potential issue. Temperature was continuously monitored using an aquarium thermometer taped to the outside of the plastic food container with the readout monitor clipped to a rucksack strap. Therefore, temperature control could be achieved by moving the case between clothing layers. 

### 2.2. Sampling

**Figure 2:**
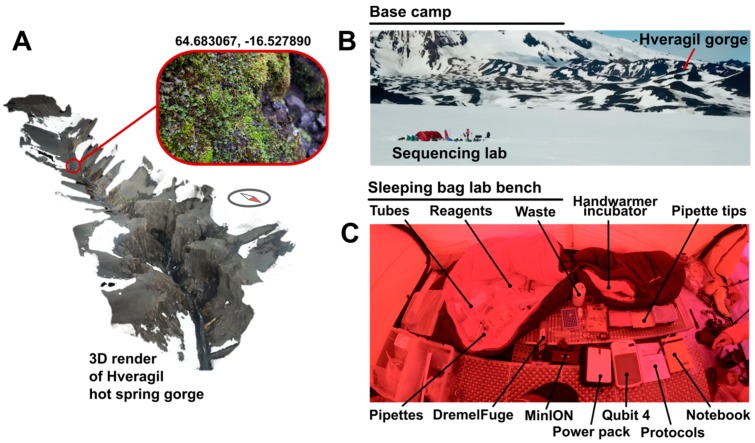
DNA extracted from Hveragil hot spring gorge. (**A**) 3D render of Hveragil hot spring gorge from a drone flight highlighting the location of sample collection (decimal degree (DD) coordinates: 64.683067, −16.527890). Render completed using footage from a Mavic Pro Drone (DJI, Shenzhen, China) and Pix4Dmapper software (Pix4D, Lausanne, Switzerland). (**B**) Landscape photograph indicating the location of sample collection “Hveragil gorge” and the base camp where DNA extraction and sequencing took place (“Sequencing lab”). (**C**) A photograph showing the set up for tent-based DNA extraction in a sleeping bag lab. Items displayed are labelled. Terralyzer for bead beating is omitted from the photograph.

Samples were obtained from the Hveragil hot spring gorge, Iceland (decimal degree (DD) coordinates: 64.683067, −16.527890) following a ~60 km traverse of the Vatnajökull ice cap (starting decimal degree (DD) coordinates 64.255293, −15.863259) by ski and sledge (Figure 2A). A metal spoon sterilised with ethanol was used to collect the sample into sterile sample bags. The sample consisted of soil approximately 3 cm under the surface of mosses and liverworts located on an overhanging rock 30 cm above the hot spring water surface (water at approximately +60 °C [17]). Samples were then transported on foot and ski at ambient environmental temperature to the tent located 3 km from the sample collection site (Figure 2B).

### 2.3. Battery-Powered DNA Extraction

DNA was extracted using the QIAGEN PowerSoil^®^ kit (Qiagen, Hilden, Germany) according to manufacturer’s instructions except for the following changes. Centrifugation steps were performed using a pre-charged Dremel™ 7750 battery-powered drill (Dremel, Prospect, IL, USA) with a 3D-printed tube holder (https://www.thingiverse.com/thing:454962). Centrifugation steps were performed on “medium” speed (level 2) for bursts of 30 s, checking for the sufficient outcome each time to minimise battery usage. PowerBead tubes were vortexed for 30 s using a TerraLyzer™ (Zymo Research, Irvine, CA, USA) with a pre-charged 12 V battery. DNA was eluted using solution C6 (sterile elution buffer) pre-heated to body temperature by holding in a gloved hand. Extracted DNA was quantified using a Qubit™ 4 fluorometer (Thermo Fisher Scientific, Waltham, MA, USA) and dsDNA HS kit according to manufacturer’s instructions powered by a PowerAdd Pilot Pro2 20 Ah power pack. During sample preparation all solutions were kept around 20 °C using hand warmers inside a closed sleeping bag. DNA concentration was performed after extraction using 0.5× Ampure XP beads (Beckmann Coulter, Brea, CA, USA). Beads were pelleted using a strong magnet and washed with 80% ethanol. Elution was carried out with deionised water. The laboratory setup is shown in Figure 2C.

### 2.4. Solar-Powered Nanopore Sequencing

Extracted DNA was prepared using the field sequencing kit LRK001 (Oxford Nanopore Technologies) according to the manufacturer’s instructions. The 80 °C water bath was achieved by filling an insulated mug (Thermos L.L.C, Schaumburg, IL, USA) with boiling water and cooling it to 80 °C with ice as appropriate. Temperature was monitored using a standard meat thermometer. The prepared DNA library was loaded onto R9.4.1 flowcells. Sequencing was powered by one PowerAdd 20000 mAh battery and two Aceyoon 20000 mAh powerbanks, all fully charged using a 90 W Solar Panel (Mobile Solar Chargers, Somerset, UK), capable of generating approximately 20 V even in moderate cloud cover, prior to any sequencing run.

### 2.5. Data Analysis

Data from Flowcell #1 was locally basecalled by offline MinKNOW (v18.12.9) (Oxford Nanopore, Oxford, UK) during the run on a Dell XPS 13 i7 laptop with 16 GB RAM and 500 GB SSD. Data from Flowcell #2 was basecalled offline after the run using Guppy (v3.0.3) (Oxford Nanopore, Oxford, UK). FASTQ files from both sequencing runs were concatenated and uploaded to Kaiju (kaiju.binf.ku.dk) for taxonomic classification upon return to civilisation due to code errors running Kaiju offline in situ. Reads were compared to the “NCBI BLAST nr + euk” reference database using the “Greedy” setting, allowing mismatches with standard parameters (minimum match length 11, minimum match score 75, allowed mismatches 5).

## 3. Results 

### 3.1. Sequencing DNA Remotely Using Solar Power

Two nanopore flowcells were taken on the expedition. A quality check was performed prior to departure and just prior to sequencing for each flowcell (Table 2).

Flowcell #1 retained 96% of the initial active pores while Flowcell #2 appeared to gain three active pores during the pre-expedition logistics and the ski traverse. These results validated our temperature control approach using body heat to maintain an above-freezing temperature during a lengthy ice cap traverse.

Within 5 h of sampling, 6.54 µg DNA was extracted in 100 µL (65.4 ng/µL) from Hveragil hot spring gorge by pooling and concentrating four separate extractions. The first sequencing run was performed on the ice cap immediately after with local basecalling activated. This was to ensure the sample contained DNA of sufficient quality for a successful sequencing run. The laptop was fully charged the day before (Day 7, Figure 1B) using power banks, which had been charged by the 90 W solar panel. This first run was started at ~8:00 p.m. on Day 8 and lasted for 5 h on laptop battery alone due to the low ambient temperature (2–8 °C). This run yielded 19,839 reads that were basecalled in real-time during the run. 

**Figure 3:**
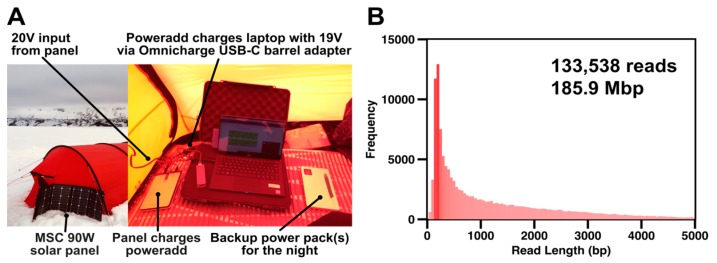
Two off-grid sequencing runs yielded almost 190 Mbp of data. (**A**) The solar power strategy for powering a laptop running Oxford Nanopore offline MinKNOW software (v18.12.9). Items shown are labelled. (**B**) Read length distribution for pooled sequencing runs. For simplicity only reads <5000 bp are shown.

To determine the maximum amount of data that a single flowcell could yield in a single solar-powered run a fresh DNA library from the same DNA extract was added to Flowcell #2. Two further additions of this DNA library were used to maximise cumulative throughput (Figure S1). Prior to the second run, three battery packs and the laptop were charged the day before (Day 10) using the solar panel. Sequencing began at 6.30 a.m. (Day 11) with local basecalling disabled and screen brightness reduced to minimise power consumption. During daylight hours power was fed into the laptop using the PowerAdd via the 90W solar panel (Figure 3A). To maximise charge efficiency the powerbank was disconnected when the laptop charged to over 80% and was reconnected when the charge dropped below 60%. This was maintained until around 6:00 p.m. when insufficient solar power could be generated to charge the powerbank. Sequencing ceased once all power was exhausted from all three powerbanks and the laptop battery at 2:00 a.m. (Day 12). Interestingly, only approximately five active pores remained at this point, indicating that sequencing was not limited by running out of power (Figure S1). This second run yielded 113,699 reads and lasted 19 h 45 min. The total sequencing time for the expedition was over 24 h and yielded 133,538 reads and 185.9 Mbp of data (Figure 3B). A large number of “short” reads (dark red, Figure 3B) result from aggressive bead beating, required to extract DNA from complex soil matrices, and the transposase step in the DNA library preparation. Despite this we still see a large number of reads extending to 5 kb in size and beyond.

### 3.2. Metagenomic Analysis

**Figure 4:**
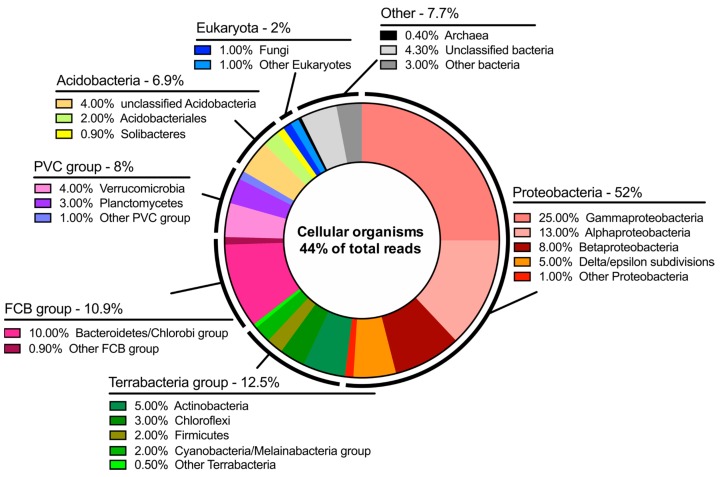
Metagenomic protein-level taxonomic identification from both sequencing runs combined. Kaiju output is shown for 44% of reads that successfully aligned to the NCBI reference database and assigned to “cellular organisms”. The relative percentage of reads assigned to “cellular organisms” of each group of organism is shown. PVC group, superphylam named after three important members Planctomycetes, Verrucomicrobia, and Chlamydiae; FCB group superphylam named after three important members Fibrobacteres, Chlorobi, Bacteroidetes.

Basecalled reads from both runs were pooled and uploaded to Kaiju for taxonomic classification. A total of 44% of the total reads (58,520) were successfully aligned to the reference database. This number is lower than was anticipated based on previous work where we have seen values between 60% and 95%. Considering the remoteness of the sampling location this is likely to represents uncharacterized microorganism species. A wide range of classes of bacteria were observed, as well as 2% eukaryotes and 0.4% archaea (Figure 4, Tables S2 and S3). Due to sample collection at the edge of a geothermal stream, 10 thermophilic bacteria were identified by taxon classification of three or more reads (*Chloracidobacterium thermophilum*, *Anaerolinea thermophila*, *Crenotalea thermop*, *Sphaerobacter thermophilus*, *Fontimonas thermophila*, *Spirochaeta thermophila*, *Symbiobacterium thermophilum*, *Schleiferia thermophila*, *Novibacillus thermophilus*, and *Pseudonocardia thermophila*). Simultaneously, 1009 reads were assigned to organisms from the Cyanobacteria phylum with the Nostocales order being most abundant (371 reads). Within Cyanobacteria morphospecies *Nostoc* (83 reads)*, Oscillatoria* (29 reads)*, Phormidium* (21 reads), and *Pseudanabaena* (9 reads) were identified which are characteristic of microbial mats in polar regions [18]. 

## 4. Discussion

In this study the successful miniaturisation of a DNA sequencing laboratory such that can be hauled by human power within a larger self-supported expedition is demonstrated. The experimental outcomes prompt some observations that may aid future similar off-grid projects. First, the DremelFuge 3D printed attachment (FormLabs clear photopolymer resin) became brittle at lower temperatures and at one point cracked during centrifugation. This both posed a safety risk and nearly derailed the DNA extraction protocol. While a backup manual centrifuge was taken [19] it too suffered from being brittle at lower temperatures. It is therefore recommended that future teams pick a more suitable resin for lower temperatures. Furthermore, there were issues keeping the Dremel drill charged using battery packs and, as such, the existing battery charge from before departure was relied upon. Since returning the Myspin 12 (Thermo Scientific, Waltham, MA, USA) has been used, which was successfully run from the PowerAdd power bank at 12 V. Due to its small footprint it is recommend future teams use this instead of the DremelFuge. 

The dichotomy of microorganisms identified at Hveragil hot spring gorge was particularly intriguing. There was a sizable number of reads assigned to both cyanobacteria characteristic of the cold polar regions and thermophilic bacteria. It is believed that this indicates the interesting microenvironment present in hot spring streams fed by meltwater from the Vatnajökull ice cap [17], resulting in a microbial population with presumably extremely diverse metabolisms. Naturally, this study demonstrates the methodology of entirely off-grid metagenome sequencing and future studies are needed to probe the microbial taxonomic distribution in this area further.

Additionally, it is worth noting that approximately 56% of the data generated could not be aligned to the “NCBI nr + euk” database. This number drops to 48% when a stringent quality threshold is applied to the reads. Therefore, it is expected most of this number represents organisms that have yet to be cultured and have their genomes characterized. While it may be expected that some of the data was from unknown organisms it is surprising that it was so high. Naturally, Hveragil gorge, on the northern edge of the ice cap, is among the most inaccessible places in Iceland. This, in combination with the interesting geochemistry of the area leads to the conclusion that 56% represents a pool of interesting organisms and metabolisms that could be later isolated and studied further. 

This entire metagenomic study was conducted off-grid, on the ice, and all while over 135 km from the nearest research facility as the crow flies. Off-grid sequencing reduced the time from sample-to-data to under 5 h compared to >days/weeks for sample collection and transport back to a research institution. In this way potential bias that may be introduced due to lengthy transport times often under suboptimal storage conditions is avoided [10]. This off-grid DNA sequencing method requires minimal infrastructure, and therefore lends itself to being integrated into a scientific program of the many expeditions that set out to the most remote corners of the polar world. This work represents a blueprint for a minimal and sustainable remote laboratory that, permitting technological advancements, could be adapted to perform other types of in situ sample extractions and downstream transcriptomics, metabolomics, or proteomics studies. By aiding the wider distribution of DNA sequencing capabilities, we hope to improve our collective understanding of the microbial communities that exist in some of the planet’s most remote environments and the role that they play in our changing climate. 

## 5. Conclusions

In this study, a microbial metagenomic analysis was conducted entirely off-grid using a laboratory small enough to be transported using human-power alone. Over 24 hours of continuous DNA sequencing using a MinION Oxford Nanopore device was conducted during a 3-week unsupported expedition on Europe’s largest icecap, the Vatnajökull in Iceland. The use of solar alone to power this sequencing effort demonstrated that microbial DNA sequencing can be conducted sustainably and repeatedly during an ongoing expedition, alongside the other physical and psychological challenges associated with survival in extreme environments. The method demonstrated here removes the need for sample transportation, and it is anticipated that this will enable researchers to study microbial populations in more remote corners of the planet, far from research stations.

## Figures and Tables

**Table 1:** Contents of the sledge-based miniaturised laboratory.

Reagents	Consumables	Hardware	
Qubit reagents	DNA Lo-bind tubes	Nanopore MinION device	Meat thermometer
Qubit standards	Qubit tubes	Tube racks	Temperature loggers (2) ^1^
AMPure beads	Sterile sample bags	Dremel drill	Magnet
80% ethanol	P1000 tips	DremelFuge adapter	Waste container
QIAGEN Powersoil kit	P200 tips	Hand-powered centrifuge	Laminated protocols
Nanopore LRK001 kit	P10 tips	Terralyser	Pen and notebook
Nanopore RAD004 kit	Nanopore flowcells	Qubit 4	90 W compact solar panel
	Gloves	USB vortex	Dell XPS 13 laptop
	Parafilm	20 Ah power packs (3) ^1^	
	Falcon tubes	P1000 pipette	
	Hand warmers	P200 pipette	
	Sterile loops	P10 pipette	

A complete DNA sequencing laboratory is miniaturised into two 9 L boxes pulled by sledge and ski across the Vatnajökull ice cap, Iceland. The contents of the two 9 L boxes comprise a functioning DNA sequencing laboratory with components listed. ^1^, the number refers to quantity.

**Table 2:** Flowcell quality check results performed prior to expedition departure on Day −4 and also immediately prior to each sequencing run on Day 8 (Flowcell #1) and Day 11 (Flowcell #2).

ID	Number of Active Pores	
	Prior to Departure Day −4	Prior to Sequencing Day 8 (#1) and 11 (#2)
Flowcell #1	1375	1320
Flowcell #2	1180	1183

## Supplementary Materials

The following are available online at https://www.mdpi.com/2073-4425/10/11/902/s1, Figure S1: Cumulative Throughput plot from sequencing run #2 shows that at the time power ran out and sequencing was stopped the cumulative throughput was essentially at a maximum, Figure S2: Read quality distributions for a standard laboratory Nanopore run and this study. Table S1: Location of items during the flight to Iceland and during the ski traverse, Table S2: Phyla taxon classification from Kaiju (see Table S3) used to construct Figure 4, Table S3: Taxanomic identification by Kaiju.
