# Supplementary material for: Entirely Off-Grid and Solar-Powered DNA Sequencing of Microbial Communities during an Ice Cap Traverse Expedition
Source: Genes (Basel). 2019 Nov 7;10(11):902. doi: 10.3390/genes10110902 (PMC6896169; doi:10.3390/genes10110902)

**Supplementary Figure S1.** Cumulative Throughput plot from sequencing run #2 shows that at the time power ran out and sequencing was stopped the cumulative throughput was essentially at a maximum.


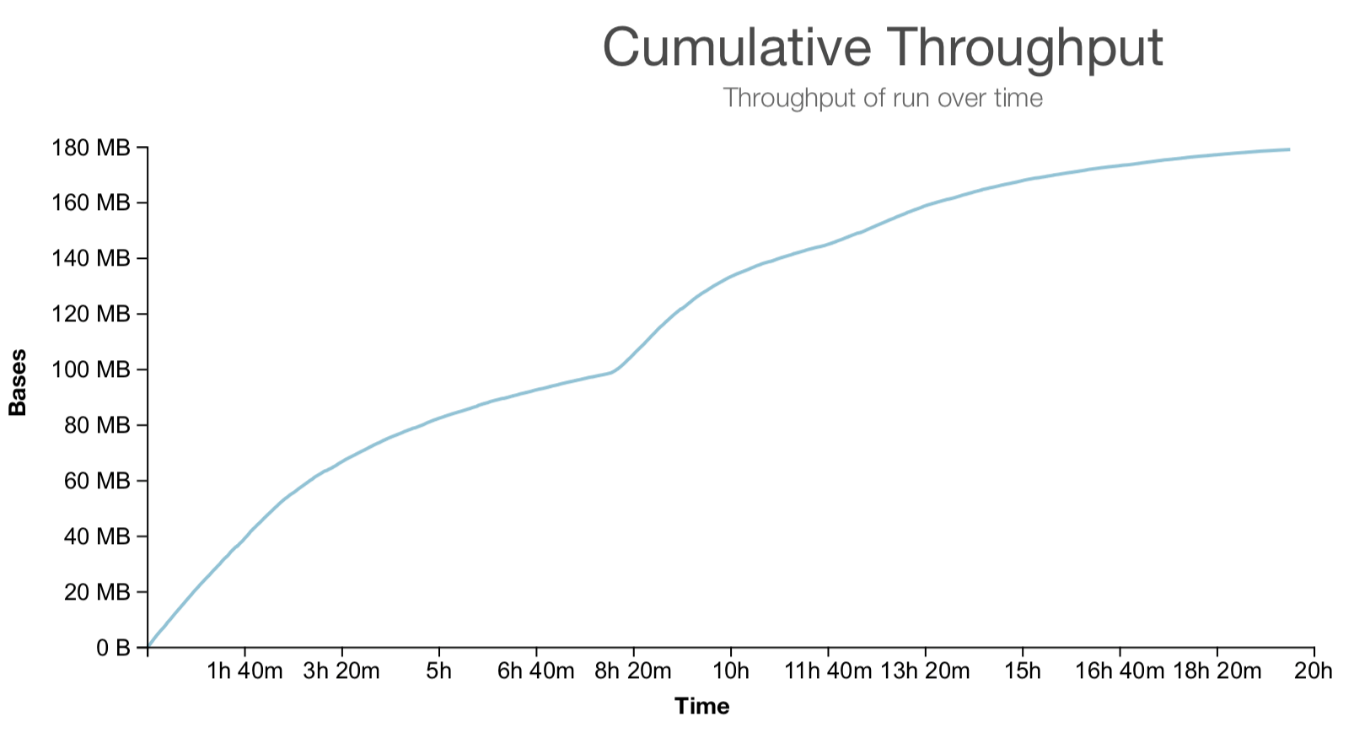


**Supplementary Figure S2.** Read quality distributions from a high throughput monoculture yeast sample run from a laboratory (top) and from this complex soil matrix on this off-grid expedition (bottom).


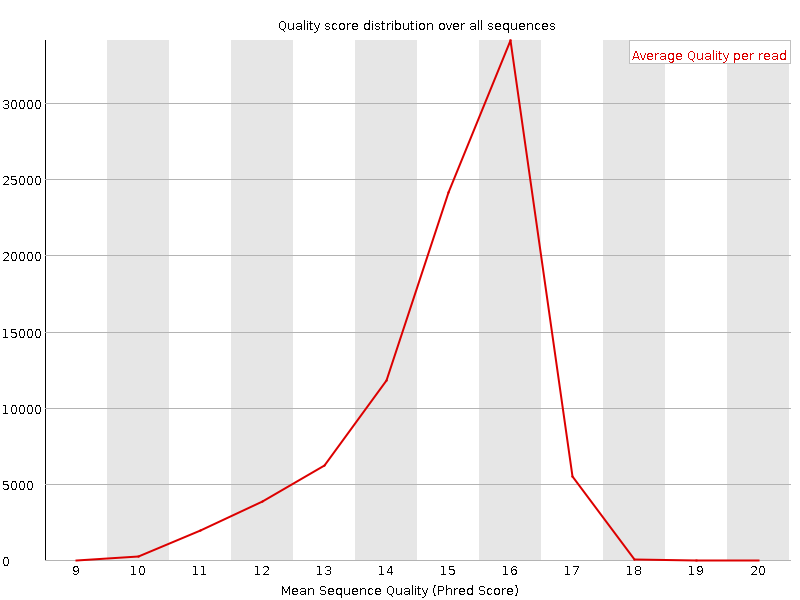


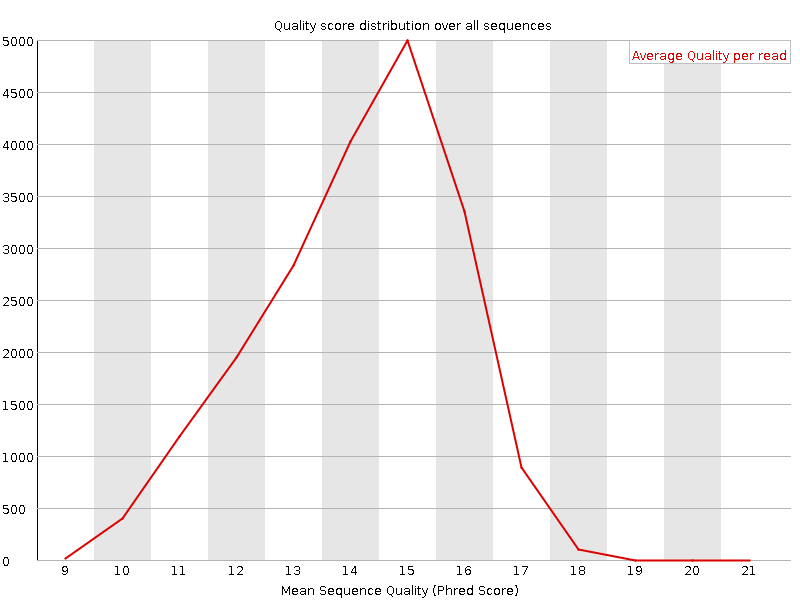

Supplement: Supplementary file 1 [file genes-10-00902-s001.zip › genes-616105-proof done-suppl/Supplementary_folder/suppl-figures.docx]
